# 1389. Nontuberculous Mycobacterial Infections of the Upper Extremity

**DOI:** 10.1093/ofid/ofab466.1581

**Published:** 2021-12-04

**Authors:** Thomas M Polveroni, Kelly Scott, Kevin J Renfree, Holenarasipur R Vikram, Carolyn Mead-Harvey

**Affiliations:** 1 Mayo Clinic Alix School of Medicine, Phoenix, Arizona; 2 Department of Orthopedics, Mayo Clinic Arizona, Phoenix, Arizona; 3 Mayo Clinic hospital, Phoenix, Arizona, Phoenix, AZ; 4 Department of Quantitative Health Sciences, Mayo Clinic Arizona, Phoenix, Arizona

## Abstract

**Background:**

Although uncommon, nontuberculous mycobacterial infections (NTMI) of the upper extremity cause significant morbidity based on their natural history, delay in diagnosis, prolonged duration of antimicrobial therapy often combined with surgical debridement, and functional loss. Herein we describe our experience with such infections.

**Methods:**

Records for adult patients from two academic, tertiary facilities with culture-proven NTMI involving the upper extremity were retrospectively reviewed. Demographic information, co-morbidities, laboratory and microbiological evaluation, management, and outcomes were extracted. Patients were analyzed based on pathogen identified and immune suppression.

**Results:**

77 patients were identified. The mean age was 59 years and 65% of patients were male. 48% reported a preceding injury, with the hand being most frequently involved (58%). 41% were considered immune compromised; 19% of them were organ transplant recipients. Mean symptom duration prior to presentation was 203 days. Mean time to culture identification was 33 days, and 25 different species of NTM were identified (subcategorized as rapid or slow growers). 77% had solitary lesions, with cutaneous/subcutaneous location as the most common site. All patients underwent surgical debridement with four undergoing amputation to control infection. 69% received combination antimicrobial therapy for a mean duration of 184 days. Immunosuppressed patients were treated with antimicrobial therapy for a longer duration (mean 243 vs 155 days). One-third of patients experienced complications and/or recurrence regardless of organism type.

**Conclusion:**

NTMI of the upper extremity is often misdiagnosed leading to significant delays in appropriate management. Knowledge of its protean manifestations and early consideration in the differential diagnosis of chronic, painful swelling of the hand or wrist, nodular or inflammatory lesions, or septic arthritis is crucial. A low threshold for surgical or biopsy with specimens sent for histopathology as well as microbiologic analysis is warranted. A combined approach with surgical debridement and prolonged combination antimicrobial therapy is necessary for optimal outcomes; however, adverse reactions from such therapy are commonly encountered.

**Disclosures:**

**All Authors**: No reported disclosures

